# Misoprostol in addition to routine treatment of postpartum hemorrhage: A hospital-based randomized-controlled trial in Karachi, Pakistan

**DOI:** 10.1186/1471-2393-8-40

**Published:** 2008-08-21

**Authors:** Nadeem F Zuberi, Jill Durocher, Rozina Sikander, Neelofur Baber, Jennifer Blum, Gijs Walraven

**Affiliations:** 1 The Aga Khan University, Dept. of Obstetrics & Gynecology, Karachi, Pakistan; 2 Gynuity Health Projects, New York, USA; 3 The Aga Khan University Hospital, Dept. of Obstetrics & Gynecology, Karachi, Pakistan; 4 Aga Khan Health Services, Dept. of Obstetrics & Gynecology, Karachi, Pakistan; 5 Secretariat of His Highness the Aga Khan, Aiglemont, France; The Aga Khan University, Dept. of Community Health Sciences, Karachi, Pakistan

## Abstract

**Background:**

Postpartum hemorrhage (PPH) remains a major killer of women worldwide. Standard uterotonic treatments used to control postpartum bleeding do not always work and are not always available. Misoprostol's potential as a treatment option for PPH is increasingly known, but its use remains ad hoc and available evidence does not support the safety or efficacy of one particular regimen. This study aimed to determine the adjunct benefit of misoprostol when combined with standard oxytocics for PPH treatment.

**Methods:**

A randomized controlled trial was conducted in four Karachi hospitals from December 2005 – April 2007 to assess the benefit of a 600 mcg dose of misoprostol given sublingually in addition to standard oxytocics for postpartum hemorrhage treatment. Consenting women had their blood loss measured after normal vaginal delivery and were enrolled in the study after losing more than 500 ml of blood. Women were randomly assigned to receive either 600 mcg sublingual misoprostol or matching placebo in addition to standard PPH treatment with injectable oxytocics. Both women and providers were blinded to the treatment assignment. Blood loss was collected until active bleeding stopped and for a minimum of one hour after PPH diagnosis. Total blood loss, hemoglobin measures, and treatment outcomes were recorded for all participants.

**Results:**

Due to a much lower rate of PPH than expected (1.2%), only sixty-one patients were diagnosed and treated for their PPH in this study, and we were therefore unable to measure statistical significance in any of the primary endpoints. The addition of 600 mcg sublingual misoprostol to standard PPH treatments does, however, suggest a trend in reduced postpartum blood loss, a smaller drop in postpartum hemoglobin, and need for fewer additional interventions. Women who bled less overall had a significantly smaller drop in hemoglobin and received fewer additional interventions. There were no hysterectomies or maternal deaths among study participants. The rate of transient shivering and fever was significantly higher among women receiving misoprostol

**Conclusion:**

A 600 mcg dose of misoprostol given sublingually shows promise as an adjunct treatment for PPH and its use should continue to be explored for its life-saving potential in the care of women experiencing PPH.

**Trial Registration:**

Clinical trials.gov, Registry No. NCT00116480

## Background

Excessive bleeding after childbirth, the leading cause of maternal deaths worldwide, has received international attention among medical and research communities for decades. A crucial component in the treatment of PPH resulting from atonic uterus is the administration of injectable uterotonics. The most commonly used agents in hospital-based settings are oxytocin and/or ergometrine. Additional medical and surgical interventions, beyond the administration of conventional uterotonics, have also been investigated as alternative and adjuvant therapy options for postpartum bleeding. The off-label use of misoprostol, a prostaglandin E1 analogue, has entered into clinical practice for this indication because of its strong uterotonic properties, and its ease in oral administration, stability at ambient temperatures, wide availability, and low cost. A 600 mcg dose of oral misoprostol has been shown safe and effective in preventing PPH [1]. Misoprostol is less effective than injectable oxytocin, and it has therefore been recommended only for prevention of PPH in settings where injectable conventional uterotonics are not available [2]. No misoprostol regimens have been proven for routine use as a stand alone or adjunct treatment for postpartum hemorrhage.

To date, only three small randomized controlled trials (RCT) of misoprostol for the treatment of PPH have been conducted [3-5]. The first RCT compared use of misoprostol (800 mcg rectally) as a first-line treatment with standard oxytocics; the latter two placebo-controlled trials assessed the additive effect of misoprostol when used in conjunction with standard oxytocics. The misoprostol regimens in these RCTs ranged from 600 to 1000 mcg and the routes of administration varied (oral, sublingual, and rectal). A trend in blood loss reduction in the misoprostol arms was shown, but the trials were not adequately powered to produce any statistically significant findings. A meta-analysis of the combined data from the two placebo-controlled trials testing misoprostol as an adjunct PPH treatment, however, does show a statistically significant reduction of blood loss of 500 ml or more after treatment [6,7]. Seven uncontrolled studies or case reports have demonstrated misoprostol's strong uterotonic effect in stopping bleeding when used as a last resort treatment in hospital-based settings [8-14]. In community-based settings, findings from an intervention trial, where Tanzanian traditional birth attendants were trained to administer 1000 mcg of misoprostol rectally as first-line PPH treatment, provided insight on the drug's potential role in PPH care [15]. Despite the promising results for misoprostol's therapeutic use as primary PPH treatment, as an additional treatment to standard oxytocics or as a last resort treatment option, the published RCTs have been small and the regimens studied have varied greatly, making it difficult to draw clear conclusions. A recent review on misoprostol for PPH noted that the current available evidence was insufficient to support the use of misoprostol for routine treatment of PPH [16]. In the absence of large randomized controlled trials with conclusive results, clinicians are left to decide on the best combination of medical interventions to control postpartum bleeding.

In Pakistan, where PPH accounts for nearly 25% of maternal deaths [17], anecdotal evidence suggests that misoprostol is often used in hospital settings. However, in a country where statistics on maternal health outcomes are largely unavailable [18], the benefits and/or risks of using misoprostol as an alternative or adjunct therapy for postpartum hemorrhage remain unknown. To establish the effectiveness of adding misoprostol to current treatment regimens, a randomized controlled trial was conducted to test a 600 mcg dose of sublingual misoprostol as an adjunct treatment at four hospitals in Karachi, Pakistan. The 600 mcg dose was selected for its demonstrated safety and efficacy in previous PPH trials of its prophylactic oral use, and the sublingual administration of misoprostol was preferred for its rapid onset of action, prolonged duration, and greater bioavailability [6]. By testing the drug's efficacy as an adjunct PPH treatment, this trial provides insight on the potential of misoprostol to treat PPH more effectively.

## Methods

The purpose of this randomized controlled trial was to ascertain whether 600 mcg of misoprostol taken sublingually provides an additional benefit to a standard oxytocin regimen for treatment of PPH. The study had a pragmatic approach with minimal interference in the current routine management of delivery. Prior to trial commencement, a meeting was jointly conducted with representatives from the participating hospitals to document routine PPH prevention and treatment protocols at their facilities, and a management protocol was developed to guide PPH treatment practices over the course of the study. The trial was approved by the Ethical Review Committee at the Aga Khan University in Karachi.

The study began in December 2005, with the last woman enrolled in April 2007 after 16 months of continuous recruitment. The study aimed to enroll 900 women over 18 months at four hospitals in Karachi, Pakistan: The Aga Khan University Hospital (AKUH) a large tertiary level hospital; and three secondary level facilities within the Aga Khan Network – Aga Khan Hospital for Women in Karimabad District, Aga Khan Hospital for Women in Garden District and Aga Khan Hospital for Women and Children in Kharadar District. Each of these hospitals has approximately 2,000 deliveries per year. Laboring women were given detailed information on the study protocol and invited to participate in the trial by trained hospital staff. A consent form (available in English and Urdu) was signed or thumb-printed by consenting women. Women with cesarean-section, gestational age less than 28 weeks at time of delivery, or not consenting were excluded from the study.

All women underwent routine active management of the third stage of labor with standard uterotonics, controlled cord traction after delivery of baby, and gentle uterine massage after delivery of the placenta. At the delivery of the anterior shoulder of baby, one of two uterotonic regimens was administered: intravenous 10 IU of oxytocin or 5 IU of oxytocin plus 0.4 mg of ergometrine given either intramuscularly or intravenously. Immediately after delivery of the baby, blood loss was collected by placing a clean fracture bedpan directly under the woman's buttocks for a minimum of one hour. Markings were written onto the bedpan to show when 500 ml had been reached. Women losing less than 500 ml were not entered into the trial. Women losing 500 ml or more were enrolled in the trial, and a clean bedpan was placed underneath their buttocks to collect blood lost after PPH diagnosis. A fresh, large perineal pad with plastic backing was positioned just below the bedpan to capture any spattering blood. Once the delivery attendant considered active bleeding to have stopped, the blood was transferred to a calibrated jar for measurement.

All women with diagnosed PPH thought to be due to inadequate uterine contraction, as per the provider's clinical judgment, were promptly given IV oxytocin as routine for PPH treatment. Women were reminded of their consent to participate in the trial and a member of study team gave each woman the pills in the next randomized study envelope and instructed her to place the tablets under her tongue, e.g. sublingually. Each study envelope contained three tablets of either misoprostol (200 mcg × 3) (Gymiso, HRA Pharma, France) or matching placebo. All women, providers, and investigators were blinded to the treatment assignments. Simultaneous to PPH treatment administration, blood collection was restarted with a clean bedpan and fresh perineal pad placed underneath the woman. Blood loss was then measured continuously until active bleeding ceased – or for a minimum of one hour. The additional blood loss after receiving PPH treatment was transferred to a calibrated jug and sealed, and all used gauzes and pads were counted and placed in a plastic bag. The plastic bag was then weighed; however, accurate use of the scales proved difficult, and these results could not be verified. Therefore, in this paper we share the validated results of total volume of blood collected.

Based on previous studies, we estimated a 10% rate of additional blood loss ≥ 500 ml [6] after receiving study treatment in the oxytocin-misoprostol arm and a rate of 16% in the oxytocin-placebo arm. To detect a difference of this size (one-sided test), we calculated that to achieve 80% power at p = 0.05, a sample size of 420 women in each arm was needed. The sample was randomized in blocks of ten, stratified by site, using a computer-generated random sequence provided by Gynuity Health Projects, New York, where the code was kept. Data were entered and cleaned in Epi Info (Epi Info, version 3.4.3). The randomization code was concealed until all data were entered and cleaned. Data analysis was conducted using the Statistical Package for the Social Sciences, version 13.0 (SPSS, Chicago, IL, USA). Using SPSS, categorical data were analyzed with chi-square tests and continuous data with t tests.

The main study outcome was to determine if the addition of misoprostol to standard PPH care reduces postpartum bleeding. The primary endpoint was measured blood loss ≥ 500 mls after PPH treatment; secondary outcomes included change in hemoglobin, side effects, need for additional interventions including blood transfusion, additional uterotonics, balloon tamponade, hysterectomy, and mean blood loss. Hemoglobin levels were measured pre-delivery upon entry into labor ward and 12–24 hours post-delivery by taking a finger prick and using a Hemocue Hemoglobin machine (HemoCue AB, Ängleholm, Sweden). Side effects were recorded by the delivery attendant as they were observed or reported. Blood loss was documented for women who consented for the study but did not experience PPH. Regular monitoring and training of delivery ward staff continued throughout duration of the trial.

## Results

Blood loss following 5,171 vaginal deliveries was collected at the four participating hospitals. The mean blood loss and postpartum hemoglobin level were 150 ml and 11.2 g/dL, respectively. Sixty-one women (1.2%) were diagnosed with PPH and randomized to receive either oxytocin plus 600 mcg misoprostol sublingually (n = 29) or oxytocin plus matching placebo (n = 32). Deviations in study protocol occurred among two women randomized to the misoprostol arm. In one protocol deviation, the patient was given only 400 mcg of study medication because the delivery ward staff questioned whether her bleeding might be due to episiotomy. The second protocol deviation involved a woman whose blood mistakenly was not collected by hospital staff. Table 1 details the baseline demographic and delivery characteristics of all study participants (n = 61). There were no significant differences between women in the two groups.

**Table 1:** Baseline characteristics among women treated for PPH

	**misoprostol**	**placebo**	
	n = 29	n = 32	RR [95% CI] or p-value

**Age (years) **mean ± sd	25 ± 5	26 ± 4	p = .291
**Parity **%(n)			
0	62.1 (18)	40.6 (13)	1.53 [.92, 2.54]
1–3	31.0 (9)	50.0 (16)	
4–8	6.9 (2)	9.4 (3)	
**Outcome of this delivery **%(n)			
Singleton, alive	93.1 (27)	96.9 (31)	.96 [.85, 1.08]
Singleton, still birth	3.4 (1)	--	
Twins, both alive	3.4 (1)	3.7 (1)	
**Episiotomy **%(n)	75.9 (22)	68.8 (22)	1.10 [.81, 1.51]
**Manual removal of placenta done **%(n)	6.9 (2)	12.5 (4)	.55 [.11, 2.79]
**Placental delivery within 5 minutes **%(n)	62.1 (18)	78.1 (25)	.79 [.57,1.11]
**Pre-delivery hemoglobin **(g/dL)			
mean ± sd	11.1 ± 1.0	10.9 ± 1.1	p = .502
range	9.1 – 13.5	8.7 – 13.0	
**Measured blood loss at diagnosis **(ml)			
mean ± sd	640 ± 139	669 ± 184^	p = .492
range	400 – 1000	500 – 1000	
**Time to diagnosis **(minutes)			
mean ± sd	37 ± 29	33 ± 24	p = .584
range	5 – 122	5 – 100	
**Use of oxytocics *prior *to study treatment **%(n)	100 (29)	100 (32)	1.0
**Use of oxytocics *after *study treatment **%(n)	100 (29)	100 (32)	1.0

^ There is one outlier excluded in which the woman was diagnosed after losing 1,750 ml.

All women had their third stage of labor actively managed as per standard hospital protocol. There were no statistical differences in the amount and route of prophylactic oxytocin administered between the two study groups. All women received oxytocin prophylaxis at the delivery of anterior shoulder of baby (IV administration 88.5%; IM administration 11.5%). The prophylactic use of ergometrine in addition to oxytocin was also standard practice at two of the four sites. In half of the deliveries (equally distributed across study arms), ergometrine was administered prophylactically (IV administration 60.0%; IM administration 40.0%) in conjunction with oxytocin.

On average, PPH was diagnosed 35 minutes following delivery after losing an average of 655 ml (± 163 ml) of blood, at which time PPH treatment was administered. There were no differences in the dose, route or choice of standard uterotonics given for PPH treatment between the two study groups. Intravenous oxytocin (bolus 5–30 units and infusion 10–80 units in 500–1000 ml saline) and ergometrine up to 0.4 mg i.v. were initial treatment measures. Oxytocin infusion was given for all PPH cases; two-thirds were given intravenous oxytocin in bolus; and only one-third of cases received 0.2–0.4 mg ergometrine i.v. Six cases (four in the misoprostol group and two in the placebo group) were given an additional dose of 200–400 mcg misoprostol either rectally or sublingually. Prostaglandin alpha F-2 was administered to one woman in misoprostol group and three in the placebo group.

There were no problems reported with sublingual administration of study medication. The study outcomes are summarized in Table 2. Due to the much lower than expected PPH rate, the intended sample size was not reached. Postpartum blood loss, drop in hemoglobin, and use of additional interventions (blood transfusion, uterine packing, and balloon tamponade) were lower in misoprostol group, but did not reach statistical significance. Fewer women in the misoprostol arm received i.v. fluids amounting to greater than 1000 ml (p = .056). Among those women receiving additional interventions (twelve in the misoprostol group; nineteen in the placebo group), total measured blood loss was significantly higher (940 ± 341 ml, p = .028) compared with women (n = 29) who did not receive any additional intervention beyond standard treatment (780 ± 176 ml). Similarly, drop in postpartum hemoglobin was significantly higher among women requiring additional interventions (2.5 g/dL (± 1.5) vs. 1.7 g/dL (± 0.9); p = .022). There were no hysterectomies or maternal deaths among study participants.

**Table 2:** Outcomes after receiving study treatment

	**misoprostol**	**placebo**	
	n = 29	n = 32	RR [95% CI] or p-value

**Postpartum blood loss after study treatment**			
	(n = 27) ^	(n = 32)	
**Total blood loss post-treatment **(ml)			
mean ± sd	175 ± 168	187 ± 207	p = .809
Range	10–700	10–900	
**Blood loss ≥ 500 ml post-treatment **%(n)	7.4 (2)	12.5 (4)	.59 [.12, 2.99]
**Postpartum hemoglobin measures**			
**Post-delivery Hb**			
mean ± sd	9.0 ± 1.4	8.7 ± 1.2	p = .291
range	5.9 – 11.3	5.9 – 10.2	
**Drop in Hb**			
mean ± sd	2.0 ± 1.1	2.2 ± 1.4	p = .614
range	0.4 – 4.2	0.1 – 5.1	
**Postpartum Hb ≥ 2 g/dL lower than pre-delivery Hb **%(n)	41.4 (12)	56.3 (18)	.74 [.43, 1.25]
**Additional interventions**			
**Amount of IV fluids given**			
500–1000 ml	75.9 (22)	53.1 (17)	
> 1000 ml	24.1 (7)	46.9 (15)	.51 [.24, 1.08]
**Blood transfusion **%(n)	17.2 (5)	18.8 (6)	.92 [.31, 2.69]
**Uterine packing **%(n)	6.9 (2)	18.8 (6)	.37 [.08, 1.68]
**Balloon tamponade **%(n)	0.0 (0)	3.1 (1)	.00 [.00, 43.0]
**Referrals for additional PPH care **%(n)	3.4 (1)	3.2 (1)	1.1 [.07, 16.9]

^ Two cases in the misoprostol arm have incomplete blood loss measurements and were excluded from analysis of measured postpartum blood loss.

Shivering and fever were more commonly reported in the misoprostol group. All other side effects were minimal (Table 3). Transient shivering was experienced by 51.7% of women receiving misoprostol compared with 6.2% in placebo group. The incidence of fever followed similar pattern to that of shivering. Eleven women (37.9%) in the misoprostol group experienced an elevated temperature of ≥ 37.5°C (≥ 99.5°F) compared with only three women (9.4%) in the placebo group. Three women receiving misoprostol characterized their fever as being severe. There was one case of high fever reported in the misoprostol group in which temperature at one hour post-treatment measured 40.1°C (104.2°F). There were no reports of severe side effects resulting in prolonged hospitalization or other adverse events in misoprostol group and all women made a full recovery.

**Table 3:** Side effects after receiving study treatment

	**misoprostol**	**placebo**	
	n = 29	n = 32	RR [95% CI] or p-value

**Nausea **%(n)			
none	93.1 (27)	93.8 (30)	.99 [.87, 1.13]
mild or moderate	6.9 (2)	6.3 (2)	
severe	--	--	
**Vomiting **%(n)			
none	93.1 (27)	96.9 (31)	.96 [.85, 1.08]
mild or moderate	6.9 (2)	--	
severe	--	3.1 (1)	
**Diarrhea **%(n)			
none	100 (29)	100 (32)	1.0
mild or moderate	--	--	
severe	--	--	
**Fainting **%(n)			
none	100 (29)	96.9 (31)	1.03 [.97, 1.10]
mild or moderate	--	3.1 (1)	
severe	--	--	
**Fatigue **%(n)			
none	100 (29)	100 (32)	1.0
mild or moderate	--	--	
severe	--	--	
**Headache **%(n)			
none	93.1 (27)	100 (32)	.93 [.84, 1.03]
mild or moderate	6.9 (2)	--	
severe	--	--	
**Shivering **%(n)			
none	48.3 (14)	93.8 (30)	
mild or moderate	37.9 (11)	6.2 (2)	8.28 [2.1. 33.1]
severe	13.8 (4)	--	
**Fever **%(n)			
none	48.3 (14)	90.6 (29)	
mild or moderate	41.4 (12)	9.4 (3)	5.52 [1.8, 17.1]
severe	10.3 (3)	--	
**Temperature at 1 hour **(Celsius)			
mean ± sd	37.4 ± .94	37.0 ± .35	p = .022
range	36.2 – 40.1	36.3 – 37.8	

## Discussion

The purpose of this trial was to explore whether 600 mcg sublingual misoprostol offers any additional benefit when added simultaneously to conventional methods for the treatment of primary postpartum bleeding. Although the study findings reported here are consistent with the two placebo-controlled trials on adjunct use of misoprostol [4,5], we were unable to measure statistical significance in our primary endpoints due to a much lower rate of PPH than expected. Our findings suggest a trend in reduced postpartum blood loss, smaller drop in postpartum hemoglobin, and fewer additional interventions among women treated with misoprostol in addition to standard oxytocics.

The use of misoprostol was not associated with a significant reduction in any of the pre-specified primary outcome measures in this study. However, the relative risk reduction of 41% of blood loss ≥ 500 ml after treatment (RR 0.59 95% CI [0.12, 2.99]) is similar to the two hospital-based placebo-controlled trials conducted in South Africa (RR 0.56 95% CI [0.21, 1.46]) and the Gambia (RR 0.58, 95% CI [0.32, 1.06]) [6]. Indeed, a combined analysis of results from this trial and the two placebo-controlled trials on adjunct use of misoprostol [4,5] confirms that misoprostol use is associated with a significant reduction of blood loss of ≥ 500 ml following treatment (RR 0.58, 95% CI [0.35, 0.95], p = .029). This confers with results from other recent meta-analyses [2,6,7].

Our study findings on measured postpartum blood loss also highlight the clinical importance of a reduction of blood loss following PPH treatment. Women who bled less overall had a significantly smaller drop in hemoglobin and did not require additional interventions, such as blood transfusion, balloon tamponade, or uterine packing, to manage their postpartum bleeding. In contrast, women who had a significantly higher total blood loss were more likely to be given additional interventions and experience a larger drop in postpartum hemoglobin. A reduction in blood loss reduces need for more invasive procedures, results in a smaller change in postpartum hemoglobin, and as a result, may prevent more severe maternal morbidity experienced by recently delivered mothers. Based on the trends in blood loss we see among women in our study given misoprostol in addition to standard oxytocics for their PPH treatment, the adjunct use of misoprostol shows great potential in improving women's health outcomes after experiencing this obstetrical complication.

As found in previous studies on misoprostol, use of the drug was significantly associated with fever and shivering. These effects, however, were described as transient and did not result in any additional complication to the women. Within the literature on misoprostol as adjunct treatment, a total of three cases of high fever ≥ 40.0°C (≥ 104.0°F) have been reported following a 1000 mcg dose of misoprostol (400 mcg sublingually + 400 mcg rectally + 200 mcg orally) [4]. This study had one case of temperature over 40.0°C in the misoprostol group. Delivery ward staff should be trained to recognize and manage cases of severe shivering and high fever to ensure proper care of the patient.

Great efforts were taken to standardize PPH care within and across the participating hospitals. Representatives from the hospitals reviewed their PPH management policies and developed a document to guide their treatment practices over the course of the study and thereafter. Despite efforts to standardize PPH care, there still existed variation in the pharmacological agents/regimens used, especially in the administration of second- or third-line therapies. For instance, six patients were given an additional dose of misoprostol beyond initial treatments. Although the hospitals in this study had policies cautioning against the use of prostaglandins due to lack of evidence, providers still relied on their use as a therapy options.

Irrespective of the variability in PPH practices, a low rate of PPH < 2% across the four participating hospitals was demonstrated in this study. This rate of PPH was validated using an objective measurement protocol with marked bedpan in over 5,000 normal vaginal deliveries. Studies on postpartum hemorrhage often find that the incidence of PPH is underreported in settings prior to objective measurement of postpartum blood loss [19-22], however this study shows just the opposite with a measured PPH rate of 1.2%. According to the 2000 Cochrane Review, the rate of PPH ≥ 500 ml is roughly 5% for women receiving prophylactic uterotonics and 12% for those not receiving uterotonics prophylactically [23]. Importantly, only two of the four studies included in the review specified the use of an objective blood measurement technique. In general, there is wide variability in reported PPH rates in the literature. As Soriano and colleagues note, PPH incidence ≥ 500 ml has been reported at 1.0%, 5.0%, 7.2%, and 14.5% in studies comparing oxytocin-ergometrine and oxytocin alone in the third stage of labor [24]. This study, using objective measurement of postpartum blood loss, confirms that a very low rate of PPH can be achieved in hospital settings following the routine practice of active management of the third stage of labor. At the same time, the low rate of PPH in this study demonstrates the difficulty in conducting hospital-based studies on relatively rare, but clinically important obstetrical complications.

The objective assessment of postpartum bleeding provided a valuable, initially unexpected, insight into the diagnosis and management of PPH in the four Karachi hospitals. Individual exit interviews with study staff revealed that they believed the use of the bedpan had corrected their "over"-estimation of postpartum blood loss. Both doctors and nurses explained that previously they had overestimated postpartum blood loss, resulting in misdiagnosis of PPH, unnecessary treatments, and prolonged hospitalization. During the study, staff was trained to diagnose PPH at 500 ml using the bedpan for objective assessment. However, PPH was diagnosed, on average, after losing nearly 650 ml, which suggests that the use of the bedpan did not lead to more prompt diagnosis at 500 ml. The timing of diagnosis, which commonly occurred around 30 minutes after delivery, also provided valuable insight on the duration of close-monitoring necessary during the postpartum period for all recently delivered mothers. Objective measures using the bedpan and Hemocue apparatus also verified that those women with greater postpartum bleeding and larger decline in postpartum hemoglobin were commonly provided with additional interventions beyond standard uterotonic treatments to control their bleeding. The use of the bedpan proved to be a valuable tool for educating delivery ward staff over the course of the study on the diagnosis and management of postpartum hemorrhage, but the value of its continued use outside a clinical trial is not yet apparent.

## Conclusion

Due to the logistical complexities of managing postpartum hemorrhage, this obstetrical complication continues to threaten women's lives, especially in facilities short-staffed or lacking uterotonics and protocols to manage PPH safely and effectively. It has been advocated that misoprostol should be available in community-based settings with limited access to conventional injectable uterotonics. This study suggests that misoprostol may also have an important role to play in hospital settings and its adjunct use should continue to be explored for its potential in quickly, safely, and effectively controlling postpartum bleeding, averting recourse to more invasive procedures, and preventing more severe maternal morbidity.

## Competing interests

The authors declare that they have no competing interests.

## Authors' contributions

NZ carried out the study, participated in its coordination and helped draft the manuscript. JD monitored the study, participated in its coordination, performed the statistical analysis and drafted the manuscript. RS carried out the study and reviewed the manuscript. NB carried out the study and reviewed the manuscript. JB contributed to design of the study, participated in its coordination, data interpretation and drafting the manuscript. GW designed the study, helped interpret data and draft the manuscript. All authors read and approved the final manuscript.

## Pre-publication history

The pre-publication history for this paper can be accessed here:


